# Targeted alpha therapy in a systemic mouse model of prostate cancer - a feasibility study

**DOI:** 10.7150/thno.42228

**Published:** 2020-02-03

**Authors:** Andreea D. Stuparu, Catherine A.L. Meyer, Susan L. Evans-Axelsson, Katharina Lückerath, Liu H. Wei, Woosuk Kim, Soumya Poddar, Christine E. Mona, Magnus Dahlbom, Mark D. Girgis, Caius G. Radu, Johannes Czernin, Roger Slavik

**Affiliations:** 1 Ahmanson Translational Theranostic Division, Department of Molecular and Medical Pharmacology, David Geffen School of Medicine at University of California, Los Angeles (UCLA), CA, USA.; 2 Department of Translational Medicine, Division of Urological Cancers, Skåne University Hospital Malmö, Lund University, Sweden.; 3 Department of Surgery, David Geffen School of Medicine at University of California, Los Angeles (UCLA), CA, USA.

**Keywords:** PSMA, targeted alpha therapy, prostate cancer, metastatic mouse model, actinium

## Abstract

^225^Ac-PSMA-617 targeted-therapy has demonstrated efficacy in 75-85% of patients; however, responses are not durable. We aimed to establish translatable mouse models of disseminated prostate cancer (PCa) to evaluate effectiveness of ^225^Ac-PSMA-617 at various disease stages.

**Methods**: C4-2, C4-2B, or 22Rv1 cells were injected into the left ventricle of male NSG mice. Disease progression was monitored using bioluminescence imaging (BLI). For treatment, mice were injected with 40 kBq ^225^Ac-PSMA-617 at one (early treatment cohort) or three weeks (late treatment cohort) post-inoculation. Treatment efficacy was monitored by BLI of whole-body tumor burden. Mice were sacrificed based on body conditioning score.

**Results**: C4-2 cells yielded metastases in liver, lungs, spleen, stomach, bones, and brain - achieving a clinically relevant model of widespread metastatic disease. The disease burden in the early treatment cohort was stable over 27 weeks in 5/9 mice and progressive in 4/9 mice. These mice were sacrificed due to brain metastases. Median survival of the late treatment cohort was superior to controls (13 vs. 7 weeks; p<0.0001) but inferior to that in the early treatment cohort (13 vs. 27 weeks; p<0.001). Late cohort mice succumbed to extensive liver involvement. The 22Rv1 and C4-2B systemic models were not used for treatment due to high kidney metastatic burden or low take rate, respectively.

**Conclusion**: C4-2 cells reproduced metastatic cancer spread most relevantly. Early treatment with ^225^Ac-PSMA-617 prevented liver metastases and led to significant survival benefit. Late treatment improved survival without reducing tumor burden in the liver, the main site of metastasis. The current findings suggest that early ^225^Ac-PSMA-617 intervention is more efficacious in the setting of widespread metastatic PCa.

## Introduction

Despite the emergence of novel therapeutic approaches including androgen deprivation therapy (ADT) 1 and investigational prostate specific membrane antigen (PSMA)-targeted alpha- and beta-particle radioligand therapies (RLT) 2-15, advanced PCa remains invariably lethal 16. ^225^Ac-PSMA-617 RLT has shown promising responses in heavily treated and naïve mCRPC patients 7. Almost 75-85% of patients experience a decline in PSA level following therapy. Early progression-free survival data show a median survival of 9 months with several patients having an enduring response 6, 7. Despite promising initial responses, ^225^Ac-PSMA-617 RLT treatment is not curative as similarly observed with ^177^Lu-PSMA RLT 6. Therefore, there is a need to better understand the responses to treatment.

The use of mouse models has aided in the discovery, mechanistic interrogation, and therapy developments for a wide range of cancers. However, the choice of the model often limits translatability. Subcutaneous xenograft models allow for easy tumor and treatment response monitoring but fail to provide information about metastatic disease. Orthotopic and systemic models reflect the clinical scenario more relevantly but are more difficult to establish and to monitor 17-22. Orthotopic primary tumors enable response assessments to different therapies. Systemic cancer mouse models, obtained via intracardiac injection, are better suited to create metastases varying in size and location 21. Yet, these models are rarely used for therapeutic efficacy studies with only few reports for RLT 23-25.

Several human cell lines are used for murine PCa models. The most commonly used PCa cell lines are the androgen-independent PC3 (derived from bone metastasis) and DU145 (derived from a brain metastasis) cells, and the androgen-dependent LNCaP (derived from a lymph node metastasis) cell line as well as derivatives of these cells 17, 26. To date, the most reported metastatic prostate cancer mouse models employ the PC3 cells or variants of these cells, including PC3-PIP, a cell line engineered to express PSMA, and PC3M a variant with higher incidence of metastases 23, 25, 27. A unique feature of the PC3 cell line variants is the formation of mandible metastases 28, 29. Whereas both PC3 and DU145 are used as androgen-independent cell lines, they do not express AR 17. We selected the following endogenously expressing PSMA and AR cells i) C4-2, a LNCaP subline, as it is PSA expressing androgen receptor (AR) mutant metastatic castration resistant prostate cancer (mCRPC) cell line that develops metastases *in vivo* 21, 30); ii) C4-2B, which are C4-2 cells collected from bone metastases, potentially providing a different metastatic pattern than the parental C4-2 cells 21, 31-33); and iii) 22Rv1, which express PSA, are AR and Tp53 mutant, and represent both primary and relapsed PCa 34, 35.

The C4-2 cell line responds well to PSMA-targeted RLT in a subcutaneous model, but disease relapses in a dose-dependent manner within weeks of treatment 36. The objective of the current experiments was to establish PCa models for studying the efficacy of ^225^Ac-PSMA-617 against different metastatic patterns and stages of the disease. To our knowledge, this is the first report evaluating the treatment efficacy of ^225^Ac-PSMA-617 in a metastatic mouse model of prostate cancer at different time points.

## Methods

### Cell culture

C4-2 and C4-2B cells were kindly provided by Dr. George Thalmann (Department of Urology, Inselspital Bern). 22Rv1 cells were purchased from American Type Culture Collection. Cells were thawed one week prior to inoculation and were maintained in Roswell Park Memorial Institute 1640 (RPMI 1640) medium supplemented with 10% fetal bovine serum (Omega Scientific) at 37°C and 5% CO_2_. Cells were monitored on a regular basis for mycoplasma contamination using the Venor GeM mycoplasma detection kit (Sigma Aldrich). The parental cells were engineered to express firefly luciferase by transduction with an amphotropic retrovirus encoding enhanced firefly luciferase followed by fluorescence activated cell sorting of transduced cells. PSMA-11 and PSMA-617 were obtained from ABX GmbH.

### Animal Studies

All animal studies were approved by the UCLA Animal Research Committee (# 2005-090). Male, 6-8 weeks old NSG mice were obtained from the UCLA Radiation Oncology Animal Core. Mice were housed under pathogen-free conditions with food and water *ad libitum*, and a 12-12 hour light-dark cycle. Veterinarian staff and investigators observed the mice daily to ensure animal welfare and determine if humane endpoints (e.g., hunched and ruffled appearance, apathy, ulceration, severe weight loss, tumor burden) were reached. The mice were inoculated with either firefly luciferase expressing C4-2 (500k cells/mouse), C4-2B (500k cells/mouse), or 22Rv1 (100k cells/mouse) cells into the left ventricle of the heart under anesthesia (2% isoflurane).

The cells were trypsinized, filtered through a 40 µm cell strainer, spun down, and resuspended in media and 50 mg/mL D-luciferin in a ratio of 1:1 to enable post-inoculation bioluminescence imaging (BLI). A successful injection was judged by both bright red blood pumping into the syringe at the end of the inoculation, as well as whole-body BLI signal 10-15 min post-inoculation. Mice with focal thoracic bioluminescence signal indicative of a failed injection, were not included in the treatment study. Disease burden and spread were monitored weekly using BLI. Mice were randomized into treatment groups based on whole-body BLI.

Mice weighed (mean±SD) 26.3±1.4 g (control), 26.4±1.4 g (early treatment), and 27.9±1.6 g (late treatment) at study start, and 23.1±1.7 g (control), 23.2±1.9 g (early treatment), and 24.7±3.3 g (late treatment) at the last time point all mice were alive. Mice were sacrificed when they exhibited rapid weight loss (>20%) and showed signs of deteriorating health due to the metastatic burden such as hunching, dehydration, and labored breathing. The overall condition of the animals was assessed using the body conditioning score 37. A drop in score from 3 (well-conditioned mouse) to 2 (underconditioned; segmentation of vertebral column evident, dorsal pelvic bones palpable) warranted euthanasia. At sacrifice, organs were inspected for visible metastases and imaged *ex vivo* by BLI for metastatic burden quantification. The organs that showed BLI signal above background, were stored in formalin and then paraffin embedded for hematoxylin and eosin (H&E) staining.

In a separate experiment, five mice were sacrificed at either 1, 3, 4, 5, or 6 weeks post inoculation for *ex vivo* metastases characterization. The organs that showed BLI signal above background, were stored in formalin and then paraffin embedded for H&E staining on four slices per organ.

### Sample size justification

A power analysis was performed using G*Power 3.1.9.4 38 to determine the sample size necessary to evaluate differences in whole body radiance between treatment groups at a given time post-inoculation. Considering a two-tailed t-test for difference in means of independent groups, with type I error rate of 0.05 and an effect size of 1.5, 80% power is achieved when using 10 mice per group.

### Intracardiac injections

In lieu of using ultrasound guidance, we marked the sternal notch, the top of the xyphoid process, and half-way in between these two spots. The needle insertion was performed slightly to the left of the midway mark on the sternum.

We drew a small bubble of air into the syringe to allow visualization of the cardiac pulse followed by drawing 100 µL of cell suspension 39. Success of left ventricular needle insertion was judged by the pulsating bright red blood in the syringe. The cells were injected slowly over a period of about 30 s. At the end of the injection, the syringe plunger was slightly pulled back to draw a minimal amount of blood into the syringe. This prevents cells spilling into the chest cavity during the needle removal and provides proof that the needle was still positioned correctly in the left ventricle. After needle removal, gentle pressure was applied to the chest of the mouse for about a minute to reduce bleeding. Mice were monitored closely for any signs of distress post-injection.

### BLI

Metastatic disease burden and spread were quantified using weekly BLI on a Xenogen IVIS 100 *in vivo* imaging system (Perkin Elmer). The mice were subcutaneously injected with 150 mg/kg D-luciferin (50 mg/mL) and imaged 15 min post injection in the supine position. Living Image software was used to quantify whole body disease burden. The data were then plotted and analyzed using GraphPad Prism 8. GraphPad Prism 8 was also used to generate survival plots and statistical analyses. The Log-rank (Mantel-Cox) test was used for survival analysis.

### ^68^Ga-PSMA-11 positron emission tomography/ computed tomography (PET/CT)

^68^Ga-PSMA-11 was synthesized by eluting gallium-68 from a ^68^Ge/^68^Ga generator (Eckert & Ziegler) with 0.1 M hydrochloric acid, trapping ^68^Ga on a cationic exchange cartridge and eluting with 5 M sodium chloride solution. 5 µg PSMA-11 in HEPES buffer were reacted with ^68^GaCl_3_ for 5 min at 95 °C. Radiochemical identity and purity were confirmed before application by radiographic thin-layer chromatography. For PET/CT, ~1.1 MBq ^68^Ga-PSMA-11 in 100 µL volume was injected into the tail vein and images were acquired 60 min later using the pre-clinical Genisys 8 PET/CT scanner (Sofie Biosciences). Attenuation corrected images were reconstructed using maximum-likelihood expectation maximization with 60 iterations. The following parameters were applied for CT imaging: 40 kVp, 190 mA, 720 projections, and 55 ms exposure time per projection. The resulting PET/CT images were analysed using the VivoQuant Imaging Software (Invicro).

### ^225^Ac-PSMA-617 Targeted Alpha Therapy (TAT)

Actinium-225 was supplied by the Isotope Program within the Office of Nuclear Physics in the Department of Energy's Office of Science. [^225^Ac]Ac(NO_3_)_3_ was dissolved in 0.1 M HCl and mixed with PSMA-617 in 1 M NaOAc containing 10 mg/mL gentisic acid resulting in a final reaction pH ~5.5. Labeling for 30 min at 90 °C provided ^225^Ac-PSMA-617 in a purity of at least 92% by radio-thin layer chromatography at a molar activity of 130 MBq/µmol. After dilution with normal saline, mice (n=10 mice/group) were injected with 40 kBq of ^225^Ac-PSMA-617 (i.v.) at either one- or three-weeks post intracardiac cell inoculation. Control mice (n=10 mice) were left untreated.

### Tissue Analysis

Tissue samples were fixed overnight in 10% PBS-buffered formalin and transferred to 70% ethanol for storage before paraffin embedding. Paraffin embedded tissues were sectioned into 4 µm slices. Sections were stained with hematoxylin (8 min) and eosin (1 min) (H&E) by the UCLA translational pathology core laboratory. For anti-human PSMA staining, paraffin-embedded sections (4 µm) were de-paraffinized and re-hydrated. Endogenous peroxidase was blocked (3% hydrogen peroxide/methanol, 10 min.). Antigens were retrieved in heated 0.01 M citrate buffer, pH 6.0 (95ºC, 25 min.). Specimens were incubated overnight at 4°C with a mouse anti-PSMA antibody (clone 3E6; 1:50, DAKO, M362029-2) in bovine serum albumin. For detection, the Dakocytomation Envision System labeled polymer horseradish peroxidase (DakoCytomation, Carpinteria) and the diaminobenzidine reaction (#BDB2004 L; Biocare Medical) were used according to the manufacturers' instructions. The sections were counterstained with hematoxylin. All slides were mounted with Cytoseal (Fisher Scientific) and scanned digitally at 20x magnification using ScanScope AT (Leica Biosystems, Vista).

## Results

### Formation of metastases

Formation of metastases was cell type dependent. Liver metastases formed in 90% of mice challenged with C4-2 cells as early as one week post-inoculation but only in 30% of mice challenged with C4-2B cells over several weeks. On the other hand, 22Rv1 cells were injected successfully in 100% of mice and all developed metastases as early as one week post-inoculation.

### Metastatic pattern

**Figure 1:**
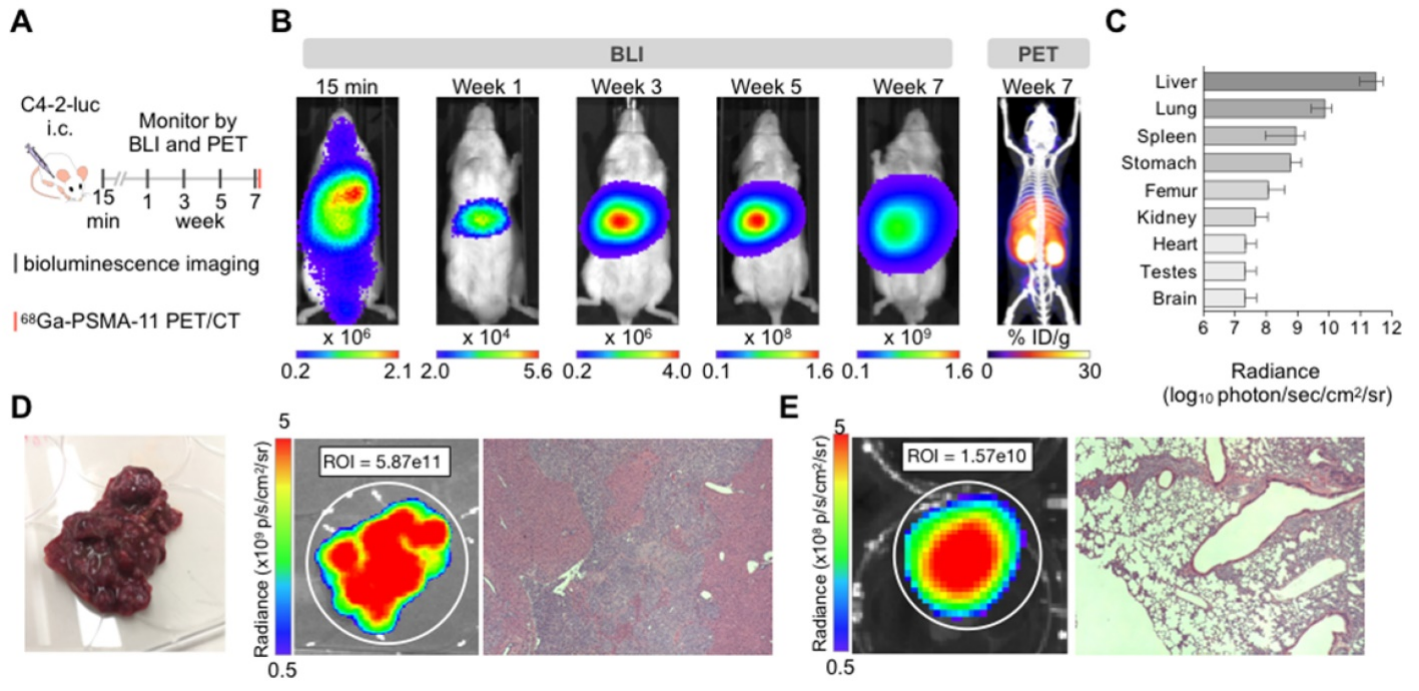
** Characterization of the C4-2 intracardiac systemic tumor model.** (**A**) Experimental design. (**B**) *In vivo* bioluminescence imaging over time of an untreated mouse inoculated by intracardiac injection and ^68^Ga-PSMA-11 PET/CT at 7 weeks. (**C**) Bioluminescence radiance (photons/second/cm^2^/sr) as means of tumor burden of single organs *ex vivo* 7-9 weeks post-inoculation (mean ± SD, n=10 mice). (**D**) Liver disease burden was visible by eye, BLI, and H&E staining in all the control mice. (**E**) Lung metastases were only visible by BLI and H&E staining.

*C4-2 cells*: Intracardiac inoculation of firefly luciferase expressing C4-2 cells led to liver micrometastases (>600 counts) after one week (**Supplementary Figure S1**). Smaller extra-hepatic metastatic sites were difficult to detect by BLI given the high intensity signal from the liver. *Ex vivo* analysis revealed other metastatic sites such as lung, spleen, stomach, bone, and brain 7 weeks post-inoculation (**Figure 1**). Time course *ex vivo* analysis revealed visible macroscopic liver lesions as early as four weeks post inoculation with the BLI signal, number, and size of lesions increasing over time (**Supplementary Figure S2**). In addition, H&E and anti-hPSMA staining revealed liver lesions otherwise non-visible by eye in 4/5 mice sacrificed at 1 week or 3 weeks post inoculation. The number of lesions and size may be underestimated due to the small number of slices. At the time of body score deterioration and subsequent sacrifice, at seven weeks multiple liver metastases were visible by eye. The presence of the C4-2 metastases was verified by ^68^Ga-PSMA11 PET/CT before sacrifice (**Figure 1B**), *ex vivo* BLI following sacrifice, and H&E staining on paraffin embedded organs (**Figure 1D**).

*22Rv1 cells*: 22Rv1 intracardiac inoculation led to the formation of visible macroscopic metastases in liver, adrenal glands, and kidney in all mice 5 weeks post-inoculation. Further lesions were detected in lung and bones by *ex vivo* BLI.

Thus, intracardiac 22Rv1 cell administration provides a robust metastatic model. However, we chose the C4-2 model for further therapy studies due to low, if any, kidney disease burden. The high metastatic burden in kidney and adrenal glands seen in the 22Rv1 model may interfere with assessment of off-target organ toxicity (**Supplementary Figure S3**).

*C4-2B cells*: The success rate for the C4-2B model was too low to robustly assess metastatic spread and tumor burden.

### ^225^Ac-PSMA-617 RLT is effective in reducing metastatic tumor burden

**Figure 2:**
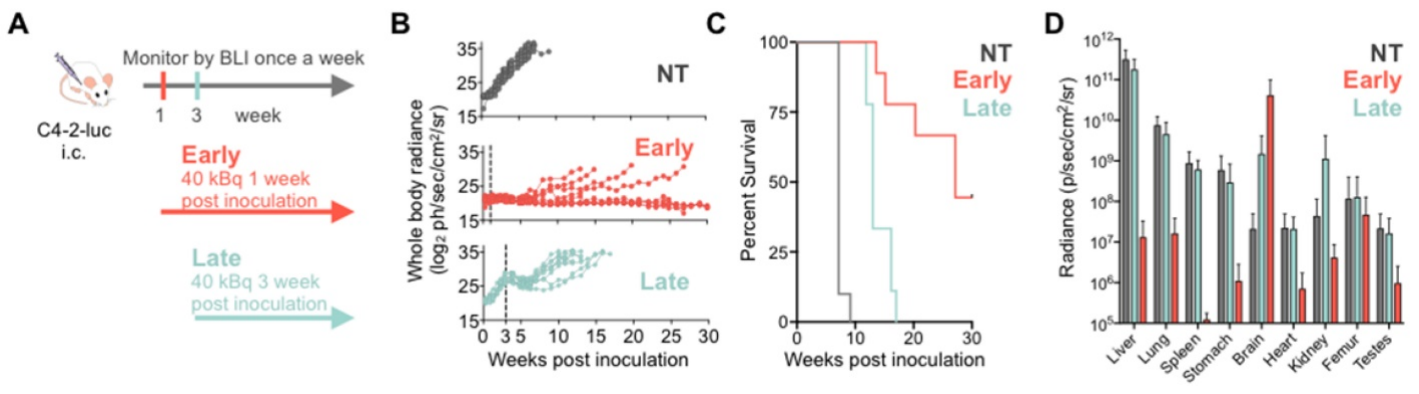
**^225^Ac-PSMA-617 RLT suppresses C4-2 tumor growth in a systemic mouse model.** (**A**) Experimental design. (**B**) Individual whole-body radiance growth curves following one cycle of 40 kBq ^225^Ac-PSMA-617 either 1 week (early) or 3 weeks (late) post-inoculation (n=10 mice per group). (**C**) Survival plot (n= 9-10 mice/treatment group). Median survival was 7 weeks (NT), 27 weeks (early treatment), and 13 weeks (late treatment), (NT vs*.* early treatment, p<0.0001; NT vs*.* late treatment, p<0.0001; early vs. late treatment, p<0.001). (**D**) Mean whole organ radiance measured *ex vivo* at time of sacrifice. Results are mean +/- SD for 10 mice for NT, 8 mice for late treatment and 5 mice for early treatment. NT - untreated.

To investigate treatment response as a function of disease burden, mice were treated with 40 kBq of ^225^Ac-PSMA-617 at either one- (early treatment cohort) or three- weeks (late treatment cohort) post intracardiac injection with C4-2 cells. This activity was chosen as it is well tolerated and efficacious in a subcutaneous mouse model (unpublished data). Early and late treatment resulted in a significant survival benefit (27 vs. 13 weeks; p<0.001). Disease remained stable in the early and late treatment groups for 8 and 3 weeks, respectively (**Figure 2**). One mouse from each group was excluded from analysis as no disease was evident on BLI.

Disease recurred uniformly after 3 weeks in all mice of the late treatment group. In contrast, disease recurred later (week 8) and only in 4/9 mice of the early treatment cohort. At termination of the study 30 weeks post inoculation, 4/9 mice in the early treatment cohort showed stable whole body tumor burden. One mouse was sacrificed at 27 weeks post-inoculation due to rapid 20% weight loss despite non-detectable whole-body disease burden. *Ex vivo*, disease was detected in the femur, brain, and liver.

Metastatic disease distribution in the late and early treatment cohorts and untreated controls was similar. All control and late treatment mice had extensive liver involvement at the time of sacrifice (**Supplementary Figure S4**). However, the late treatment cohort had more extensive brain involvement than the untreated controls (**Figure 2D**). All mice exhibited sudden and rapid weight loss (15-20%) around their time of sacrifice (**Supplementary Figure S5**). The early treatment cohort had significantly more extensive brain involvement as compared to both late treatment cohort and the untreated controls (**Figure 2D, Supplementary Figure S6**) maybe due to the fact that mice were sacrificed later.

**Figure 3:**
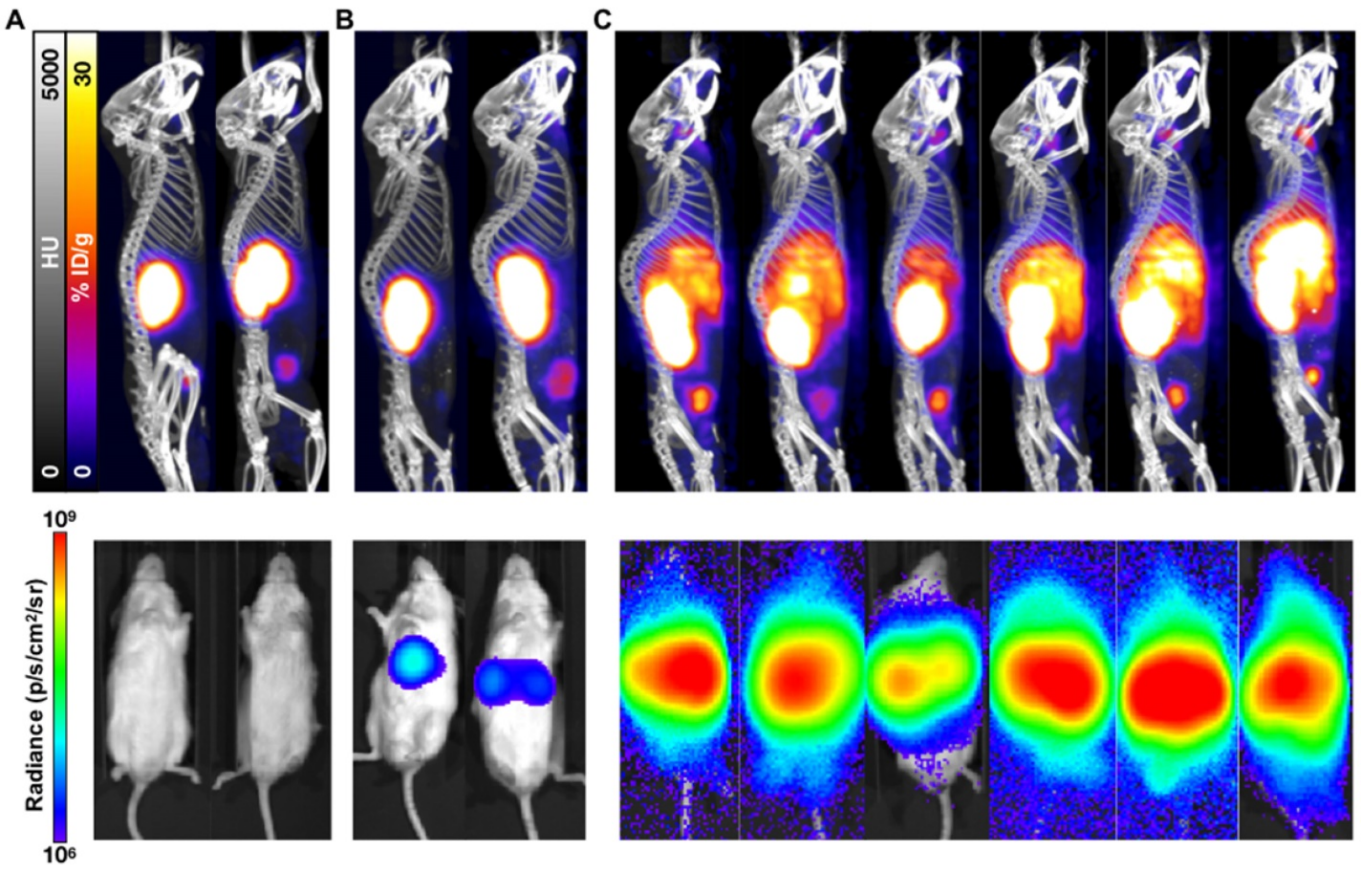
**PET/CT and BLI characterization at 7 weeks post-inoculation of C4-2 cells.** Representative mice treated with 40 kBq of ^225^Ac-PSMA-617 at (**A**) 1 week (early treatment cohort), or (**B**) 3 weeks (late treatment cohort) post-inoculation, and (**C**) untreated control mice were imaged using ^68^Ga-PSMA-11 and BLI. Images show detectable liver disease in control mice by both PET/CT and BLI, but only by BLI for the late treatment cohort. No detectable disease was observed for the early treatment mice.

**Figure 4:**
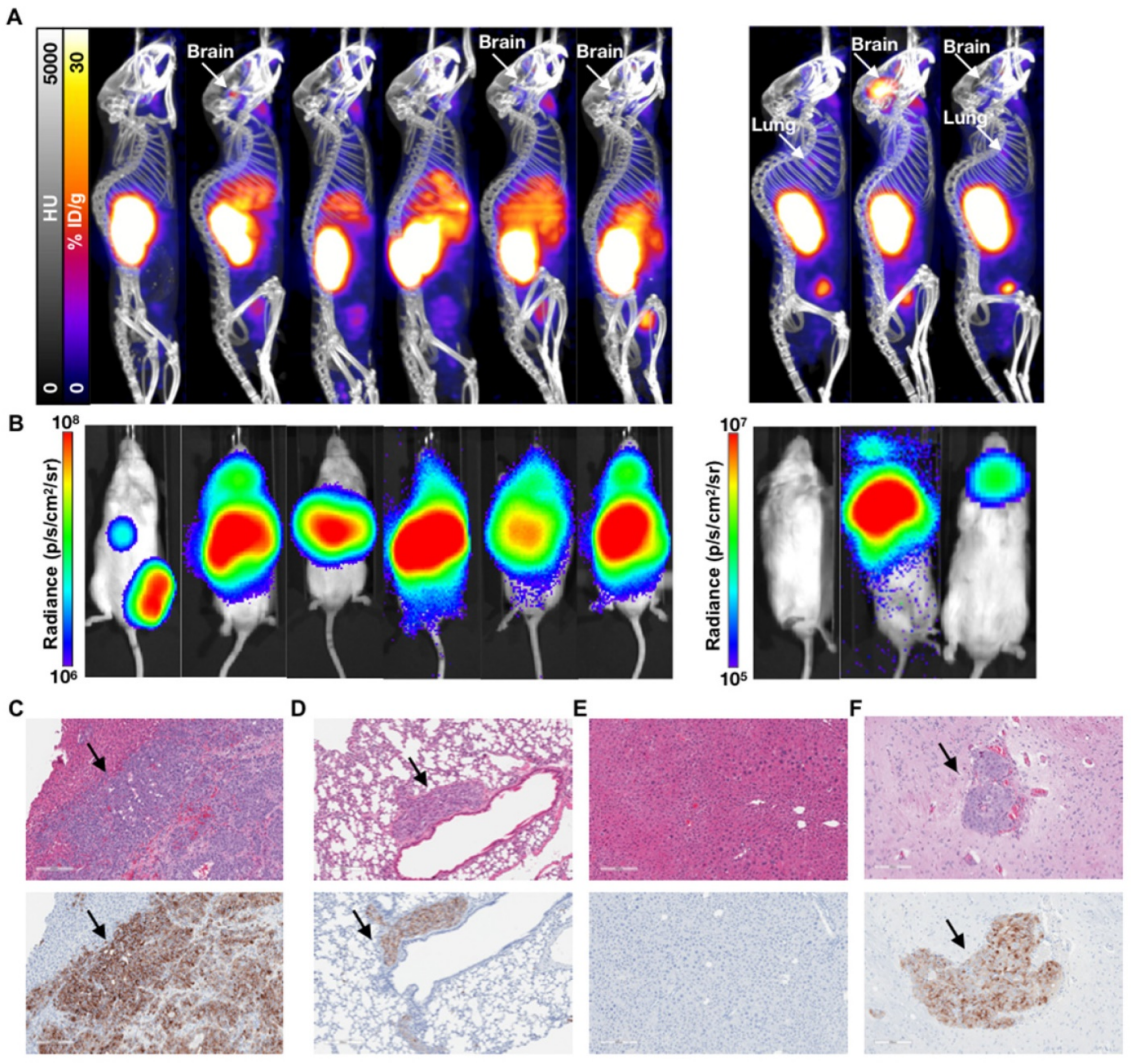
** Characterization of the 1 week (early) 3 week p.i. (late) treatment groups.** (**A**) ^68^Ga-PSMA-11 PET/CT imaging of six mice from the 3 week (late) p.i. treatment group at 80 days post treatment and three mice from the 1 week (early) p.i. group at 144 days post treatment. (**B**) Corresponding *in vivo* bioluminescence images of the mice at 78 and 141 days post treatment, respectively. Most mice showed various degrees of liver diseases, with one mouse showing focal uptake in the left femur (not visible by PET/CT) and four mice showing significant brain disease. All metastases sites were confirmed by *ex vivo* BLI. (**C**) H&E and anti-hPSMA IHC staining of liver and (**D**) lung slices from a late treatment mouse. Black arrows indicate metastases. (**E**) H&E and anti-hPSMA IHC staining of liver and (**F**) brain from a representative early treatment mouse. The depicted H&E and anti-hPSMA sections have a 10x magnification and a scale bar of 200 µm. Black arrows indicate metastases.

While not as sensitive as BLI for metastatic disease burden detection, PET/CT imaging confirmed the differential tumor burden in the control versus treated mice (**Figure 3**). ^68^Ga-PSMA-11 PET/CT imaging was used to confirm PSMA expression over time as BLI cannot be used for this purpose. PET/CT images acquired 7 weeks post-inoculation showed high hepatic disease burden in control mice, with no detectable metastases in the early and late treatment cohorts. Brain lesions were detected in 3/6 late treatment as well as in 2/3 early treatment mice imaged by ^68^Ga-PSMA-11 PET/CT 12 weeks post-inoculation (**Figure 4**).

## Discussion

Herein, we describe the development of a mCRPC mouse metastatic model suitable for monitoring PSMA-targeted RLT at various disease stages. To achieve this, we performed intracardiac inoculations of C4-2, C4-2B, and 22Rv1 human PCa cell lines. Disease burden and metastatic spread were monitored using bioluminescence imaging and ^68^Ga-PSMA11 PET/CT. We found C4-2 to be the most suitable cell line as it led to the formation of consistent visceral tissue and bone metastases. In addition, the C4-2 cells recapitulate late-stage mCRPC characteristics such as androgen-independence, high PSMA expression, and PSA secretion. Whereas 22Rv1 cells produce metastases similarly to C4-2, they also form adrenal and kidney metastases thus making it harder to assess possible treatment-induced kidney toxicity. Still, the PSMA expression, PSA secretion, and mutations in AR, TP53, and BRCA1, make 22Rv1 a suitable PCa metastatic model. To date, our efforts to establish a reliable C4-2B metastatic model have not been successful. The C4-2B cells may be better suited for intratibial inoculations where the aim would be to assess TAT purely in bone metastases 39, 40.

Overall, 22Rv1, the faster growing cell line as xenograft, engrafted better with fewer cells required at inoculation. However, the pattern of metastases could not be predicted from the cell line characteristics. For example, the C4-2B cells are very similar to the C4-2 parental cells, yet they did not result in the formation of spleen metastases. In addition, despite being isolated from a bone metastasis, the C4-2B cells did not result in a significant increase in bone metastases in our model.

In a proof-of-concept treatment study, we show that treatment in the C4-2 metastatic model at a lower disease burden results in better outcomes. However, brain metastases impede longer survival. This could be due to insufficient radiation dose delivered to the brain through poor blood brain barrier penetration, or the dose being mostly delivered to the liver, the organ with the highest disease burden (sink effect). The significant survival benefit from the early treatment supports the current efforts to treat patients with PSMA-targeted RLT earlier and before other treatment options such as chemotherapy and androgen deprivation therapy 7, 41. Our model suggests that treating at a micrometastatic stage, before disease could be detected by PET/CT imaging, could improve patient outcomes. Treatment at a higher tumor burden is still efficacious with an overall median survival of 13 weeks, as compared to 7 weeks without treatment. Similar to controls, the late treatment cohort succumbed to high hepatic disease burden.

The inability to sufficiently reduce the liver disease may be due to insufficient radiation dose delivered to the tumor, or metastatic lesions being too large for treatment with short-range alpha particles.

The C4-2 metastatic model does differ in the pattern of metastases formation (primarily liver, but also in lungs, spleen, bone, and brain) from typical mCRPC patients who first develop lymph node and bone metastases later followed by visceral tissue metastases. However, patients with visceral metastases, especially lung and liver, have poor prognosis and do not respond well to ^177^Lu-PSMA-617 beta-targeted therapy 10, 42, 43. The high liver and lung metastatic tumor burden in our model makes it a useful representation of patients with visceral metastases. Our results suggest that ^225^Ac-PSMA-617 could delay progression even in patients with high visceral metastatic load. More data is required to show whether ^225^Ac-PSMA-617 can lead to better clinical responses than beta particle targeted therapy. To date, ^225^Ac-PSMA-617 is mostly used in patients with extensive bone disease since its shorter range in tissue will lead to lower bone marrow toxicity than seen with beta particle targeted therapy. It is possible that a combination of both alpha- and beta-targeted therapy will yield improved outcomes in patients with both visceral and bone metastases.

Despite encouraging initial clinical results with TAT, responses are not durable 2-7. One potential reason for relapse or poor RLT treatment response in patients is insufficient radiation dose delivered to tumor lesions. Our model can be used to model dosimetry to different lesion locations and sizes for improved dose delivery. If dosimetry-calculated activities are too high, TAT-induced toxicity could be diminished through a dual isotope combination 44.

One limitation of our study is the lack of patient-derived xenograft (PDX) models which are difficult to establish for PCa. Further studies are warranted to test the viability of a systemic PDX PCa model. Our model also does not recapitulate the impact of the immune system since human cell lines derived cancers can only be used in immune-deficient mice. Nevertheless, we believe this systemic model is more clinically relevant than subcutaneous xenograft models for treatment regimen efficacy and characterization studies.

## Conclusions

In summary, we characterized the efficacy of PSMA-targeted alpha therapy in a metastatic PCa mouse model. Our data highlight that the treatment time has an impact on therapeutic effectiveness. Both one- and three-week treatment time points used in this study emphasize effectiveness of PSMA-targeted therapy even against visceral tissue metastases, which are more difficult to treat and are associated with a worse prognosis. In addition, the promising efficacy of ^225^Ac-PSMA-617 at a low disease burden, detectable by BLI, but not yet by PET/CT, suggests potential benefits for treating at a minimal residual disease stage. Further studies are warranted to compare differences between alpha- vs. beta-targeted therapy and to develop optimized combination therapies of radionuclides and pharmacological inhibitors modulating DNA damage response and repair pathways. For such studies, the use of a systemic model is preferred over used subcutaneous models.

## Supplementary Material

Supplementary figures.

## Abbreviations

BLI: bioluminescence imaging
CT: computed tomography
FACS: fluorescence activated cell sorting
H&E: hematoxylin and eosin
i.c.: intracardiac
i.v.: intravenous
mCRPC: metastatic castration resistant prostate cancer
NT: untreated
PCa: prostate cancer
PET: positron emission tomography
p.i.: post-inoculation
PSMA: prostate specific membrane antigen
RLT: radioligand therapy
TAT: targeted alpha therapy
TLC: thin layer chromatography
