# Supplementary material for: Targeted alpha therapy in a systemic mouse model of prostate cancer - a feasibility study
Source: Theranostics. 2020 Feb 3;10(6):2612–20. doi: 10.7150/thno.42228 (PMC7052903; doi:10.7150/thno.42228)
Supplement: Supplementary file 1 — Supplementary figures. [file thnov10p2612s1.pdf]

Supplementary Material

Targeted alpha therapy in a systemic mouse model of prostate cancer – a feasibility study

Andreea D. Stuparu[1#], Catherine A.L. Meyer[1#], Susan L. Evans-Axelsson[2], Katharina Lückerath[1], Liu H. Wei[1], Woosuk Kim[1], Soumya Poddar[1], Christine E. Mona[1], Magnus Dahlbom[1], Mark D. Girgis[3], Caius G. Radu[1], Johannes Czernin[1], Roger Slavik[1*]

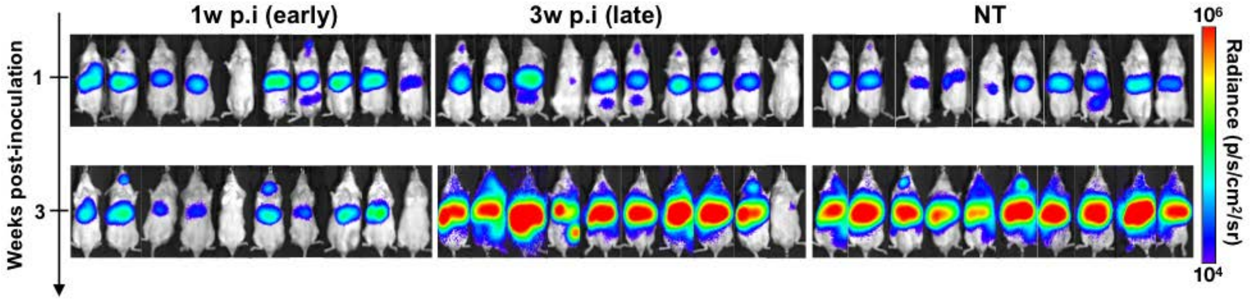

Figure S1. **Bioluminescence images at the time of treatment of NSG mice that were inoculated with C4-2-luc cells intracardially.** All, but one mouse in the 1 week (early) treatment group showed significant tumor burden at the time of treatment. One mouse in the 3 weeks (late) treatment group did not show consistent significant disease burden over the time of treatment. These two mice were excluded from analysis.

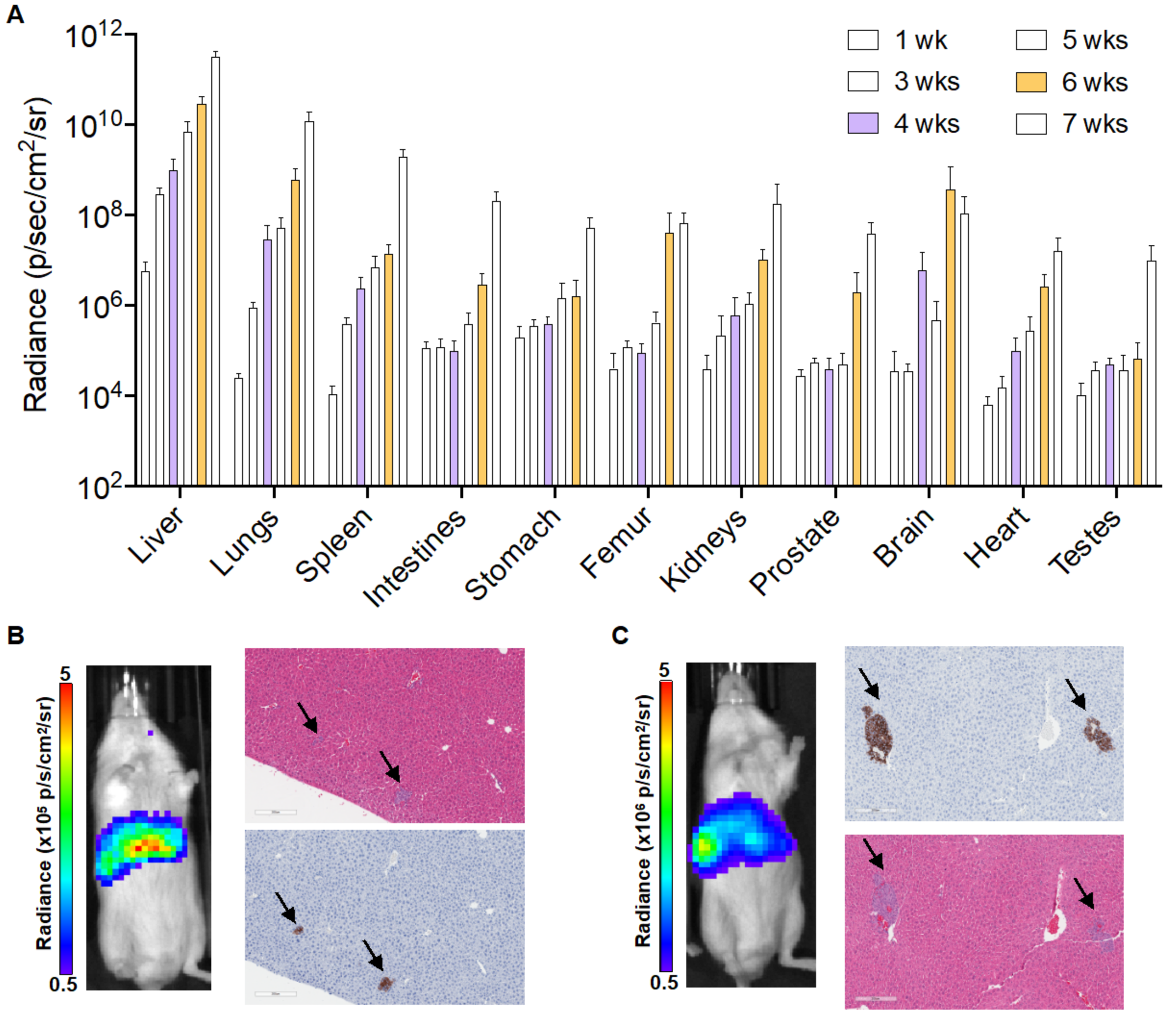

Figure S2. Metastatic distribution over time. (**A**) *Ex vivo* bioluminescence imaging shows increasing disease burden over time. Liver tumor burden is detectable by whole-body BLI, anti-hPSMA IHC, and H&E staining at 1 week (**B**) and at 3 weeks (**C**), respectively. The depicted H&E and anti-hPSMA sections have a 10x magnification and a scale bar of 200 µm. Black arrows indicate metastases.

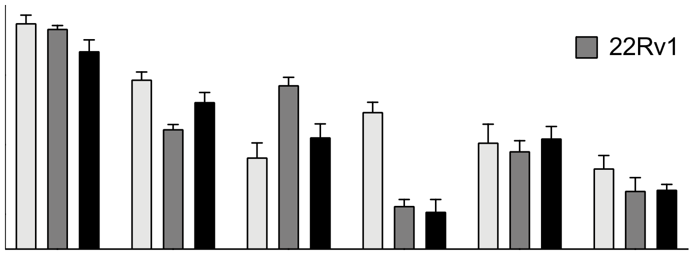

Figure S3. Comparative metastatic distribution in the C4-2, 22Rv1, and C4-2B models. *Ex vivo* bioluminescence imaging shows high disease burden in the liver and lungs in all three models. The 22Rv1 model shows 100-fold greater radiance in the kidneys as compared to the C4-2 model. Both the 22Rv1 and the C4-2B model show decreased tumor burden in the spleen as compared to C4-2.

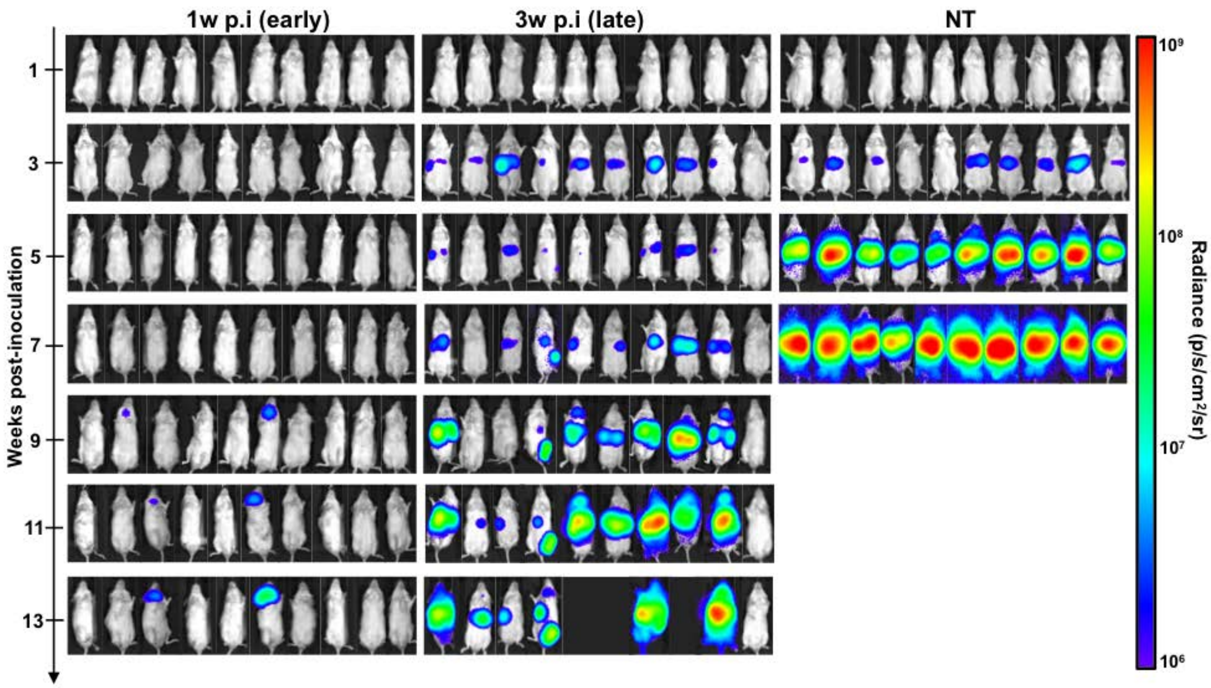

Figure S4. **Bioluminescence images of NSG mice that were inoculated with C4-2-luc cells intracardially and treated with 40 kBq of ^{225}Ac-PSMA-617 at either 1 week (early) or 3 weeks (late) post-inoculation.** Treatment reduces tumor burden and increases survival as compared to untreated (NT) control mice.

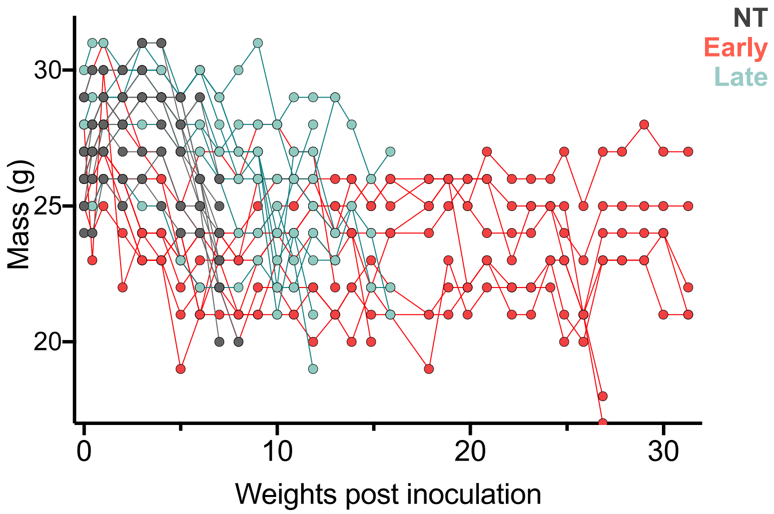

Figure S5. **Mouse weights during the treatment study.** Weights were recorded on a weekly basis. All mice exhibited sudden and rapid weight loss (15-20%) around their time of sacrifice.

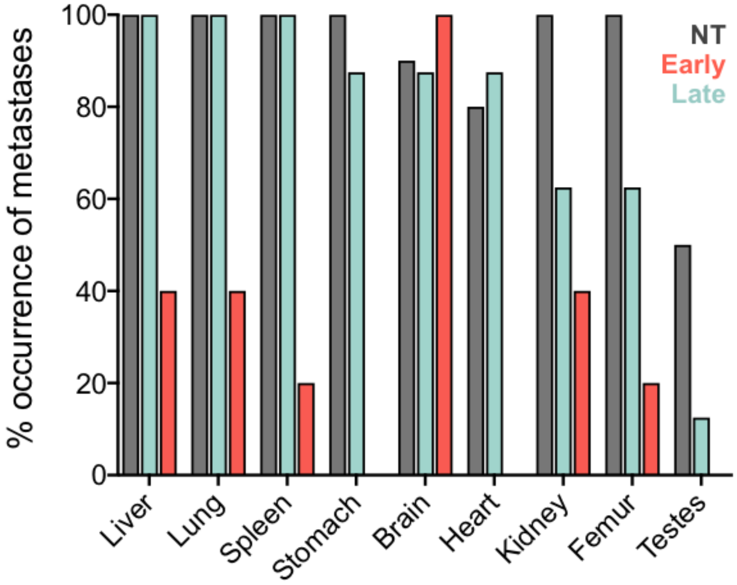

Figure S6. Metastases percent occurrence. Early treatment cohort mice exhibited fewer liver and higher brain metastases as compared to untreated control and late treatment cohort mice. Late treatment cohort mice exhibited a similar metastatic pattern to untreated controls. The percent occurrence of metastases was determined using a 600 counts BLI threshold for the *ex vivo* organs as significant.
